# Supplementary material for: Control of scabies in a tribal community using mass screening and treatment with oral ivermectin -A cluster randomized controlled trial in Gadchiroli, India
Source: PLoS Negl Trop Dis. 2021 Apr 16;15(4):e0009330. doi: 10.1371/journal.pntd.0009330 (PMC8081337; doi:10.1371/journal.pntd.0009330)
Supplement: S1 Table — (DOCX) [file pntd.0009330.s002.docx]

**S1 Table: Distribution of severity of scabies (based on number of skin lesions) at**

**baseline and two-month follow-up evaluation.**

| **Severity of scabies (number of lesions)** | **Intervention** | | **Usual care** | |
| --- | --- | --- | --- | --- |
|  | Baseline n (%) | Two-month Follow-up  n (%) | Baseline n (%) | Two-month Follow-up  n (%) |
| **Mild** (<10) | 39 (42.4) | 11 (34.4) | 50 (43.1) | 83 (65.4) |
| **Moderate** (11-49) | 32 (34.8) | 17 (53.1) | 41 (35.3) | 32 (25.2) |
| **Severe** (≥50) | 21 (22.8) | 4 (12.5) | 25 (21.6) | 12 (9.4) |
| **Total** | 92 (100%) | 32 (100%) | 116 (100%) | 127 (100%) |
