# Supplementary material for: Control of scabies in a tribal community using mass screening and treatment with oral ivermectin -A cluster randomized controlled trial in Gadchiroli, India
Source: PLoS Negl Trop Dis. 2021 Apr 16;15(4):e0009330. doi: 10.1371/journal.pntd.0009330 (PMC8081337; doi:10.1371/journal.pntd.0009330)
Supplement: S2 Table — (DOC) [file pntd.0009330.s003.doc]

**S2 Table: Symptoms reported by scabies patients in 12 villages at baseline evaluation**

**(n=208).**

| **Suffering of Scabies cases** | **n (%)** | **Duration of symptoms in days-**  **median (range)** | **Duration of symptoms in days-**  **mean (SD)** |
| --- | --- | --- | --- |
| **Itching** | 208 (100) | 30 (2 – 365) | 37 (49) |
| **Sleep deprivation** | 114 (54.8) | 15 (2 – 120) | 34 (41) |
| **Skin Sore** | 4 (1.9) | 17 (2 – 90) | 32 (41) |
