# Supplementary material for: Control of scabies in a tribal community using mass screening and treatment with oral ivermectin -A cluster randomized controlled trial in Gadchiroli, India
Source: PLoS Negl Trop Dis. 2021 Apr 16;15(4):e0009330. doi: 10.1371/journal.pntd.0009330 (PMC8081337; doi:10.1371/journal.pntd.0009330)
Supplement: S3 Table — (DOCX) [file pntd.0009330.s004.docx]

**S3 Table: Adverse drug reactions due to ivermectin and permethrin in the intervention**

**arm.**

| **Adverse drug reactions** | **Ivermectin**  **n=241** | **Permethrin**  **n=55** |
| --- | --- | --- |
| **Itching** | 11 (4.5 %) | 6 (11.0 %) |
| **Dizziness** | 8 (3.3 %) | 0 |
| **Nausea** | 6 (2.5 %) | 0 |
| **Headache** | 2 (0.8 %) | 0 |
| **Burning Sensation over skin** | 2 (0.8 %) | 0 |
| **Rash** | 0 | 1 (1.8 %) |
| **Total** | **29 (12.1 %)** | **7 (12.7%)** |
