# Supplementary material for: Control of scabies in a tribal community using mass screening and treatment with oral ivermectin -A cluster randomized controlled trial in Gadchiroli, India
Source: PLoS Negl Trop Dis. 2021 Apr 16;15(4):e0009330. doi: 10.1371/journal.pntd.0009330 (PMC8081337; doi:10.1371/journal.pntd.0009330)
Supplement: Attachment — Submitted filename: Review feedback.pdf [file pntd.0009330.s008.pdf]

Review feedback:

Lines 126 to 134: The authors are requested to elaborate further on sample size calculation, with information about the rationale behind taking the prevalence of scabies to be 7%, and the size of the cluster considered while calculating DEFF from the given ICC of 0.2

Lines 132 to 134: The authors are requested to rephrase the sentence.

Lines 344 and 345: Kindly rephrase the statement – it seems that the risk of persistence of scabies is more in the intervention arm from the mentioned sentence.

Lines 387 and 388: The claim of improved compliance with ivermectin needs to be bolstered with a reference.

Lines 392 to 395: The sentence needs re-framing to a passive voice

Line 417: "Sixty nine (59.4%) refered scabies patients…" – It should be sixty or fifty-nine.

Lines 450 and 451: An elaboration on the hypothesis would be appreciated.
