# Supplementary material for: Control of scabies in a tribal community using mass screening and treatment with oral ivermectin -A cluster randomized controlled trial in Gadchiroli, India
Source: PLoS Negl Trop Dis. 2021 Apr 16;15(4):e0009330. doi: 10.1371/journal.pntd.0009330 (PMC8081337; doi:10.1371/journal.pntd.0009330)
Supplement: Attachment — Submitted filename: Reviewer_Response_PNTD_D_20_01303.doc [file pntd.0009330.s007.doc]

**Response to Reviewer Comments**

**Ref:** PNTD-D-20-01303

**Manuscript title:**  Control of scabies in a tribal community using mass screening and treatment with oral ivermectin -A cluster randomized controlled trial in Gadchiroli, India

We sincerely thank the editor and reviewer for their helpful suggestions and comments. We have revised the manuscript as per the suggestions and comments of reviewers. The revised manuscript has improved in content and presentation. A point to point response of each of the reviewer’s comment is given below. The revised manuscripts (track file and clean file) along with reviewer comments are attached in the revised submission.

***Responses to reviewer’s comments (Method section)***

**Comment**:  It is not clear what the definition of tribal is in the context of this study. Please describe what makes this population unique.

**Response**: We have now provided the definition of tribal in the methods section. These people belong to one of the scheduled tribes described in the Constitution of India. We have also provided additional information about various major tribes in the district and the major tribe (Gond) in the 48 villages where the study was conducted. Tribal people remain geographically, socioeconomically and politically marginalized which calls for more research on the health issues affecting these people. Thank you.

**Comment**: Line 116 states that there are 48 tribal villages where the organization works and these are the villages that were included in the random selection for the study. It is not clear however, what proportion of the total tribal population these 48 villages make up in Gadchiroli. Please include, the total population of Gadchiroli, total tribal population and total population within the 48 villages.

**Response**: We have now specified this in the methods section. 40% of the total population of the district (10,72,942) is tribal (about 415000). The tribal population of these 48 villages is 12188. So about 3% of the total tribal population of the district lives in these villages.

**Comment**: Line 118, please give a range and average of village sizes included in the random selection.

**Response**: The village population varies from 100 to 737 with an average of 254. We have now stated this in the manuscript.

**Comment**: Line 123, please justify why scabies prevalence at 2 months was selected as the primary outcome. Itch can persist up to 3 months post successful treatment (Romani et al., 2015, NEJM) and has been used in this study as part of the diagnostic criteria. Residual symptoms may persist to produce false positives at 3 months.

**Response**: We did not use residual itching as an endpoint. The diagnosis by physician at two months included presence of lesions of scabies defined as pruritic papules in typical anatomical distribution. Most of the studies evaluating the effect of the ivermectin and permethrin have used time periods ranging from 2 to 4 weeks. Two recent reviews have summarized this -

Thadanipon K et al. Efficacy and safety of antiscabietic agents: A systematic review and network meta-analysis of randomized controlled trials. J Am Acad Dermatol. 2019 May;80(5):1435-1444.

Rosumeck S, Nast A, Dressler C. Evaluation of Ivermectin vs Permethrin for Treating Scabies-Summary of a Cochrane Review.

JAMA Dermatol. 2019 Jun 1;155(6):730-732.

We chose an end point of two months for logistical reasons as the study needed visiting and repeating the survey in these 12 villages.

**Comment**: Delete “in the intervention arm” in line 126.

**Response**: Thanks. We have deleted these words in the revised manuscript.

**Comment:**  Line 130, 6 clusters per arm is very few for this study design. Please justify your reasoning for selecting this sample size.

**Response:** We choose sample size using the formula given by Hayes and Bennett (Hayes RJ, Bennett S. Simple sample size calculation for cluster-randomized trials. Int J Epidemiol 1999; 28: 319–26) to estimate of sample size for cluster-randomised trial.

We assumed a between-cluster correlation coefficient of 0.2 (k=0.2) as there were no data available from prior studies. Considering, the prevalence of scabies in the usual care arm of 7% (previous experience from field practice area), loss to follow up of 20%, the study needed 6 villages per arm (average population of 250 per village) to detect 50% reduction in the prevalence of scabies with 80% power and 95% confidence (α=5%).

| **Sample Size Calculation for Scabies Trial** | | | | | | |  |  |  |  |  |  |  |  |
| --- | --- | --- | --- | --- | --- | --- | --- | --- | --- | --- | --- | --- | --- | --- |
| **S.No** | **Z1** | | **Z2** | **Proportion of control (∏0)** | **Proportion of Test positivity in intervention (∏1)** | **n** | **k** | **(z1+z2)²** | **∏0(1-∏0)/n** | **∏1(1-∏1)/n** | **[∏0(1-∏0)/n]+[∏1(1-∏1)/n]** | **k²(∏0²+∏1²)** | **(∏0-∏1)²** | **Number of villages / group [C]** |
| 1 | 1.96 | | 0.84 | 0.07 | 0.035 | 250 | 0.2 | 7.84 | 0.0002604 | 0.0001351 | 0.0003955 | 0.000245 | 0.001225 | 5.0992 |
| Considering non-response rate 20%, at cluster level the total villages in each arm are rounded to 6 village. | | | | | | | | | | | |  |  |  |

**Comment:** MSAT stands for mass screening and treatment, suggest using this correct expansion but describing what it means for this particular study.

**Response:** MSAT in our study imply mass screening by CHW, confirmation of scabies by physician for the screen positive persons, followed by treatment of scabies cases with two-doses of ivermectin (200 µg/kg) with a gap of 14 days and single-dose ivermectin (200 µg/kg) for all house-hold contacts. We have incorporated the description MSAT in our study context in the manuscript as per your suggestion. Thank you.

**Comment:** Please elaborate if these 48 villages that were included for random selection were comparable, was there a variation in population size per cluster?

**Response:**

As discussed above, out of 48 villages in the field practice area of SEARCH, six villages had a population less than 100 and excluded, 12 villages were selected randomly from the remaining 42 villages. The population of village size varies from 100 to 737 with an average 254. These points have been incorporated in method section.

**Comment:** Line 133, what about in the control arm? Usual care with resource supplementation from a study seems to lead to reductions in scabies prevalence.

**Response:** In our study, we provided resource supplementation making permethrin 5% lotion available in the MMU clinic. In control arm, the diagnosed scabies cases and their contacts were advised to visit MMU or a primary health centre (PHC) to avail the treatment. This has been described in the method section of manuscript.

**Comment:** Line 144, what is the definition for minors in this study?

**Response:** Persons with age less than 18 years is considered minor. We have incorporated in revised version of the manuscript. Thank you.

**Comment:** Can you explain why there were these exclusion criteria for screening in lines 145-147? As this is a community health centred project, it is important to consider all members of the community as everyone is a potential source of transmission.

**Response:** There were two broad groups which were excluded. Those who were already receiving ivermectin or permethrin were excluded because including them would have led to double treatment. The other category excluded were those who were seriously ill or have allergy to the study medicines. They were excluded for the potential risk of serious adverse event.

**Comment:** Can the authors please add information about is ivermectin available in this area?

**Response:** Availability of ivermectin in this area is sparse. The ivermectin tablets were available in some of shops at a distance of 50 kms or more. We have added a line in the methods section to highlight this.

**Comment:** Line 149, can the authors describe in more detail about the criteria by which cases of scabies were diagnosed?

**Response:** Scabies was diagnosed by physician with the following standard definition “presence of more than one pruritic, inflammatory papule with a typical anatomical distribution involving web spaces of fingers, hands, wrists, elbows, knees, trunk, groin or ankles”. This is already stated in the methods. Thank you.

**Comment:** Line 152, it would be helpful to have a flowchart that outlines the two-phase process and the interval of time between them, which is unclear.

**Response:** The physician visited the screen positive individuals within 7 days of mass screening. The typical time gap between CHWs screening and physician visit was 2-3 days. We have now included a line to make this clear in the methods section. Thank you.

**Comment:**  Line 154, describe how valid is the two-phase approach, this is a critical issue

**Response:** Two phase approach using screening by community health workers followed by evaluation by a physician is very commonly used in the field of public health to reduce the work load of more trained personnel. The screening questionnaire inquired about current itching, presence of papules or pustules and the location of lesions. We wanted to ensure that field workers should not miss any case of scabies during screening. To evaluate this, a physician evaluated ~ 5% of randomly selected individuals who were found to be screen-negative after assessment by the CHW. Out of 153 individuals who were screen-negative on assessment by the CHWs, all 153 did not have scabies after evaluation by a physician. Those with either itching or presence of papules or pustules at sites typical for scabies were considered screen positive. In the baseline survey, 700 individuals from both the arms were screen positive for either itching, papules or pustules in the sites typical for scabies. Out of these 208 (29.7%) were diagnosed with scabies.

**Comment :** Lines 162-164 belongs in results

**Response:**  We try to incorporate this in result section of manuscript. However, the coherence of results section was disturbed. Since this information pertains to the performance of the questionnaire we have kept it in the methods. Thank you.

**Comment :** How did you classify people that were residents of the village? How long would they have to live there? How did you record visitors?

**Response:** Individuals staying more than 6 months in the study villages were considered as the residents. SEARCH has a population register which is updated annually so residents and visitors could be separated.

**Comment:** Was permethrin applied as directly observed therapy for those that were ineligible for ivermectin?

**Response:** It was not possible to provide directly observed topical permethrin application due to privacy issues. However, the household members were counselled by CHWs about the procedure of permethrin application.

**Comment****:** Please explain why only a single dose of ivermectin was provided to the household members of a scabies case?

**Response:**. House-hold contacts of scabies cases who did not have scabies were given single-dose of ivermectin. Two-doses of ivermectin are recommended for those with confirmed diagnosis of scabies by various guidelines. There are no clear guidelines regarding the dose of ivermectin for household contacts so the options were not providing ivermectin or providing single-dose. We chose single-dose treatment for house-hold contacts as they may have potential risk of developing scabies.

**Comment:** Lines 190-191, was gentian violet paint the only treatment provided for those with impetigo? Is this standard of care in this region? Was a referral to medical facilities offered to receive antibiotics?

**Response:** Topical gentian violet 0.5% remains effective in treatment of impetigo (Maley AM et al, Exp Dermatol. 2013 Dec; 22(12): 775–780, Roderic S et al, BMJ 2004) and studies have shown benefits of gentian violet against methicillin resistant Staphyllococcus aureus (Okano M et al. Int J Dermatol. 2000 Dec;39(12):942-4). It is not commonly used in the Western world which might create a concern regarding its efficacy. We have used this agent for more than 25 years in the field with good results. The option of referral for receiving oral antibiotic in case gentian violet did not work was available, either from the MMU or via referral to nearby PHC or other doctors in the area.

**Comment :** Under the “Usual care arm” section, please specify if usual care included treatment of contacts

**Response:** Yes. This has been included under “Usual care arm” section as per your suggestion. Thank you.

**Comment:** Line 196, when were cases referred in the usual care arm? Was this during the screening visit?

**Response:** The referral was done after the confirmation of diagnosis by the physician and not after screening visit. Thank you.

**Comment:** Line 202 “patients being toxic” is unclear, suggests changing to “patients showing systemic signs and symptoms of infection” or be clear about the signs and symptoms that met criteria for referral to the rural hospital.

**Response:** The suggested modification has been included in the revised manuscript. Thank you.

**Comment:** Lines 207-213, the study provided treatment of scabies cases found at 2 months. This is a critical issue as this changes the intervention to baseline and 2 months rather than a single intervention for measuring the 12 month outcome.

**Response:** In the follow-up at two months, scabies cases in the intervention area received ivermectin or permethrin while those in the usual care area received 5% topical permethrin provided by the MMU of SEARCH. The treatment of scabies cases at 2 months could not be omitted because of ethical concerns.

**Comment****:** Line 223-225, more detail is required for the calculation of cluster-adjustment in the analysis.

**Response:** We have now used random effects logistic regression models to account for clustering and provided more information on this in the methods section. Based on revised analysis, we have also updated the results accordingly. Thank you.

**Comment:** How was consent obtained for adults that lacked capacity to provide informed consent?

**Response:** For the adult participants that lacked capacity to provide informed consent, the written informed consent was obtained from the head of household. Thank you.

***Responses to Reviewer’s Comments (Result section)***

**Comment:** Line 248- There seems to be a big difference in the sample size of the intervention and control group by over 30%, did this have an effect on the results? This issue should be included in the discussion.

**Response:**  We evaluated the effect of the intervention on the prevalence of scabies which is calculated by dividing the number of scabies cases by the population. Therefore, we do not think that difference in the population size in two arms is a concern. We have now included a line in the results section to highlight that the total population of two arms was different.

**Comment:** Please add 95% confidence intervals to your prevalence figures- was the increase in 12-month prevalence from baseline in the control group significant?

**Response:** 95% confidence intervals were presented in the tables. We have now also added 95% confidence intervals to prevalence figures in the text of the results section. Yes, 12-month prevalence from baseline in control group was significantly higher.

**Comment:** It would be good to describe the age-specific prevalence of scabies and impetigo that you found as these findings might shed some light on the resurgence of scabies at 12 months.

**Response:** Age-specific prevalence of scabies in the intervention and enhanced usual care arms are provided below. There is an increase in the prevalence of scabies among individuals of 15 years or below at the end of 12 months in both intervention and enhanced usual care arm. It may suggest there was an influx of new scabies cases from the students who study in residential boarding schools (age group <=15 years).

| **Intervention Area** |  |  |  |  |  |  |  |  |  |  |
| --- | --- | --- | --- | --- | --- | --- | --- | --- | --- | --- |
|  |  | Baseline | | | 2 Months' Follow-up | | | 12 Months' Follow-up | | |
|  | Age | Total | Diagnosed with Scabies | % Scabies | Total | Diagnosed with Scabies | % Scabies | Total | Diagnosed with Scabies | % Scabies |
|  | 0-5 | 120 | 13 | 10.8 | 115 | 3 | 2.6 | 116 | 13 | 11.2 |
|  | 6-10 | 88 | 12 | 13.6 | 99 | 5 | 5.1 | 95 | 9 | 9.5 |
|  | 11-15 | 57 | 9 | 15.8 | 77 | 2 | 2.6 | 76 | 12 | 15.8 |
|  | 16-20 | 118 | 11 | 9.3 | 114 | 7 | 6.1 | 92 | 7 | 7.6 |
|  | 21-25 | 117 | 6 | 5.1 | 116 | 4 | 3.4 | 118 | 5 | 4.2 |
|  | 26-30 | 107 | 8 | 7.5 | 117 | 2 | 1.7 | 116 | 7 | 6.0 |
|  | More than 30 | 486 | 32 | 6.6 | 499 | 7 | 1.4 | 500 | 28 | 5.6 |
|  | Missing | 1 | 0 | 0.0 | 0 | 0 | - | 0 | 0 | - |
|  | Total | 1094 | 91 | 8.3 | 1137 | 30 | 2.6 | 1113 | 81 | 7.3 |
|  |  |  |  |  |  |  |  |  |  |  |
|  | **Control Area** |  |  |  |  |  |  |  |  |  |
|  |  | Baseline | | | 2 Months' Follow-up | | | 12 Months' Follow-up | | |
|  | Age | Total | Diagnosed with Scabies | % Scabies | Total | Diagnosed with Scabies | % Scabies | Total | Diagnosed with Scabies | % Scabies |
|  | 0-5 | 171 | 11 | 6.4 | 166 | 21 | 12.7 | 173 | 25 | 14.5 |
|  | 6-10 | 90 | 6 | 6.7 | 100 | 15 | 15.0 | 125 | 30 | 24.0 |
|  | 11-15 | 61 | 9 | 14.8 | 54 | 10 | 18.5 | 72 | 19 | 26.4 |
|  | 16-20 | 145 | 14 | 9.7 | 133 | 13 | 9.8 | 128 | 23 | 18.0 |
|  | 21-25 | 162 | 12 | 7.4 | 159 | 6 | 3.8 | 168 | 17 | 10.1 |
|  | 26-30 | 137 | 17 | 12.4 | 132 | 10 | 7.6 | 137 | 17 | 12.4 |
|  | More than 30 | 664 | 48 | 7.2 | 685 | 52 | 7.6 | 696 | 81 | 11.6 |
|  | Missing | 2 | 0 | 0.0 | 1 | 0 | 0.0 | 2 | 0 | 0.0 |
|  | Total | 1432 | 117 | 8.2 | 1430 | 127 | 8.9 | 1501 | 212 | 14.1 |
|  |  |  |  |  |  |  |  |  |  |  |

Prevalence of impetigo was very low so tabular presentation does not add much value.

**Comment:** Line 307-309, is the comparison appropriate?

**Response:** Yes. We compared the prevalence in the two arms (intervention arm vs enhanced usual care arm) at two and twelve months.

***Responses to reviewer’s comments (Conclusion section)***

**Comment:** Line 371-372, this is not the main reason why this study is important, it is important because it is the first trial using MSAT - please re-write this sentence to reflect that

**Response:** Thank you for highlighting this. We have modified the discussion as per your suggestion.

**Comment:** Lines 377-380, it is valuable that the end prevalence was lower in the intervention arm compared to control, but ultimately, the prevalence at the end was the same as baseline in both groups, please explain why you think that the intervention was successful when there was no improvement in prevalence by 12 months.

**Response:** Our comparison here is between the intervention arm and the control arm at 12 months rather than that between the baseline and 12 -month prevalence in the intervention arm. This is the benefit of a controlled trial design over before and after design. The findings highlight that there is year to year variation in the prevalence of scabies which has also been our clinical experience in the past 10 years in this area. We have provided month-wise data on the number of scabies cases treated by MMU for 2 years in response to a subsequent comment which shows month to month variation. Data from the trial suggests that the prevalence increased in both the arms at 12 months follow up but it was lower in the intervention arm compared to the enhanced usual care arm- *7.3% (95% CI 5.9-9.0) in the intervention arm compared to control arm 14.1% (95% CI 12.5-16.0).*

**Comment:** Line 391-392, please specify, at which time point prevalence fell by 55%.

**Response:** At the end of 12 months. We have now specified this in the discussion.

**Comment:** Line 393-395, yes that is right. Perhaps this paper speaks to the importance of subclinical cases as later spreaders. However, please discuss how a lower baseline prevalence might have been a factor as well.

**Response:** We have now discussed this in the discussion section. Thank you.

**Comment:** Such large fluctuations of scabies prevalence after the intervention in both the intervention and control (standard care group) make the results difficult to interpret. Besides migration and not treating subclinical infestation, is there any data that might demonstrate seasonal fluctuations?

**Response:** The large fluctuation in the prevalence of scabies reflects the real life situation in this area. The data on number of cases of scabies treated by the MMU in 48 villages over a period of 2 years before the trial highlights seasonal fluctuations.

**Comment:** Line 399, this is a simplification. There are more strategies that the two that are stated here

**Response:** We have modified the sentence considering your suggestion. Thank you.

**Comment:** Line 402-403, also island population versus non-island population

**Response:** The argument of island population versus non-island population is incorporated. Thank You.

**Comment:** Line 404-405, this is true, please also refer to WHO informal consultation on a framework for scabies control meeting report published in July 2020

**Response:** Thanks, we have included the suggested reference.

**Comment:** Line 408 “apriorily”- what does this mean?

**Response:** In the trial protocol, we planned to implement mass drug administration with ivermectin if the prevalence of scabies turns out to be more than 10% in the community and mass screening and treatment if the prevalence is <10% in the baseline survey. “apriorily” means in our trial protocol it was pre-defined which strategy we will choose based on the prevalence of scabies at baseline. More details about the trial intervention are published in the clinical trial registry of India where the trial was registered. http://www.ctri.nic.in/Clinicaltrials/pmaindet2.php?trialid=16516.

**Comment:** Lines 413-415, please rephrase, this sentence is very confusing

**Response:** The sentence is rephrased. Thank you

**Comment:** Line 415-417, it is a strength of this study that this data was collected however it is missing in the results and methods section, please include it there. How long was presentation to health facilities following the visit monitored for? How was it monitored?

**Response:** We have included the referral treatment related information in both method and result section as follows-

Method: The treatment data of referred scabies patients were extracted from the monthly clinic register of the MMU. The availed treatment history was also obtained from the participants during their follow-up visits in the trial period.

Result: In the control arm forty percent (47/116 participants) of referred scabies cases availed treatment in the MMU or a PHC where scabies cases as well as their contacts received treatment.

**Comment:** Lines 420-421, please elaborate what environmental factors in the population make it highly conducive to scabies transmission?

**Response:** We have included the environmental factors like rainfall, humidity, cold weather which are conducive to scabies transmission. Thank you.

**Comment:** Line 435-436, “In a study in Australia, 95% of scabies cases were attributed to environmental factors”, delete this

**Response:** We have deleted this sentence. Thank you.

**Comment:** Is there any reason that explains higher scabies prevalence at 12 months than baseline in the control group? Surely the community would be more aware of the condition with the study visits.

**Response:** In the community where we conducted this study, a large number of children from the villages included in the trial study are enrolled in **residential schools** where scabies is quite common. When these children visit their families in the villages they often become a source of infection. Similarly, family members visiting from other villages could be a potential source of scabies. These potential explanations for higher prevalence of scabies at 12 months is already discussed.

**Comment:** Line 465-466, this is a critical point and needs to be expanded.

**Response:** We have expanded discussion on this point as per your suggestion. Thank you

**Comment:** Line 467, please define what “moderate’ scabies prevalence is here.

**Response:**  Thanks, we have now defined moderate in the revised version.

**Comment:** Line 473, was data on acceptability collected? The authors cannot claim this if no acceptability data was collected

**Response:** We have not collected data regarding acceptability therefore, we have dropped the word “acceptability” as per your suggestion.

**Comment:**  Lines 474-477, this is an excellent way to end

**Response:** Thanks for your compliment.

***Responses to reviewer’s comments (Summary and General Comments)***

**Comment:**

Reviewer #1: PNTD-D-20-01303
The primary objective of this study was to compare the impact of selective treatment of persons with scabies and their household contacts with ivermectin vs standard care (referral and topical treatment) on scabies prevalence 2 months after intervention. They found that ivermectin was superior to standard care for this and for several secondary outcomes. While innovation and originality are not strengths of this study, the results are clear and the report is well written. However, I believe that the study suffers from serious design flaws. These and other issues are listed below:

1. The study compared two very uneven interventions (selective treatment with ivermectin for cases and household contacts vs. usual standard of care (topical therapy), which for 60% of cases and contacts was no care whatsoever. This should not have been a surprise to the investigators who knew that residents of the study area have limited access to medical care (PHC or MMU). Is it scientifically valid to compare a DOT intervention with an intervention that required active participation by participants? Consider this analogy: Two interventions were offered to drowning people. One group were provided with floatation devices, while the other group was instructed to swim 500 meters to an assistance center where flotation devices would be provided on request. Which intervention worked better?

**Response:** It is important to understand the contexts while evaluating such healthcare delivery interventions in low resource settings. The key question from a healthcare delivery perspective for us was whether referral to a pre-existing service (MMU) would work or an additional component of Directly Observed Treatment (DOT) would be more beneficial. This is especially important in tribal areas as the healthcare seeking of tribal people is heavily influenced by cultural factors. If screening followed by referral would have worked well, then there is no need for DOT which needs extra training of CHWs. In the villages where the study was conducted the usual way a patient with scabies seeks care is if she/he feels that the discomfort is affecting occupational or household work or lesions look disfiguring for children. Symptoms are tolerated for very long periods. The patient will either go to a PHC or the MMU. In the enhanced usual care arm, we provided additional service in the form of active screening and referral (a prompt to seek care) which the data showed worked in 40% of cases. It was quite possible that a higher percentage of cases could have gone to a physician after the referral. It was also possible that the participants could have declined receiving a DOT from the CHW as tribal people rely heavily on traditional medicines. The trial helped us address this question. In the enhanced usual care arm, the study ensured that the permethrin 5% which is the standard of care for scabies is available with the MMU. The participants who were diagnosed with scabies in the enhanced usual care arm were also counselled regarding the availability of treatment and timing of visit of MMU.

**Comment:** More than 10% of participants were less than 5 years of age, while ivermectin is only approved for ages 5 and above (or 15 kg).

**Response:**  We have provided permethrin 5% local application for the children having indication for treatment as ivermectin is contraindicated among less than 5 years (<15 kg). This has been described both in registered protocol as well as manuscript.

<http://www.ctri.nic.in/Clinicaltrials/pmaindet2.php?trialid=16516>.

**Comment:** Methods should include a definition for "impetigo".

**Response:** Thanks, we have included the definition of impetigo in the methods section. Impetigo is defined as the presence of a papular, pustular, or ulcerative lesion surrounded by erythema.

**Comment:** It seems that outcomes of the two interventions were compared based on ITT and not corrected for crossovers due to pregnancy, age, etc. That is not a problem, but the authors should clearly state whether this was an ITT analysis. For the scabies clearance analysis, they should also provide a per protocol analysis.

**Response:**  Thanks for your comment. We have included in method section that ITT analysis was used. We are not clear what did reviewer mean by “scabies clearance analysis”.

**Comment:** Were study team members who assessed adverse events shortly after treatment and presence/absence of scabies at 2 months and 12 months blinded with regard to treatment history? If not, what measures were taken to reduce possible bias in ascertainment of AEs or assessment of scabies?

**Response:** The study team members who assessed presence or absence of scabies at 2 and 12 months, and adverse events were blinded with regard to treatment history. We have included this in revised manuscript. Thanks for your suggestion.

**Comment:** The authors suggest that more studies are needed to determine when to implement MDA vs. selective treatment for scabies. Given the high cost of the two-step screening process and somewhat disappointing results in terms of new cases and persistent scabies at 2 and 12 months in the intervention area, I think that the Discussion should cite these results as a strong argument for MDA. It is clear that many cases of scabies are sub-clinical, and others might be missed in the first round of screening by community health workers. Some cases identified by the primary screen were not present for secondary screening or treatment, and that probably diminished the impact of the ivermectin intervention. It is likely that MDA would have provided superior results to selective treatment.

**Response:** We respect the reviewer’s opinion. We think that cost-effectiveness analysis comparing two approaches will settle the choice of MDA vs MSAT in communities with relatively lower prevalence of scabies (<10%). Thank you.

**Comment**

Reviewer #3: This is an important study, it’s the first published paper on a mass screening treatment approach for the control of scabies and as such, provides novel evidence. Its overall well designed within a cluster randomized approach and it is well written overall with appropriate discussions and conclusions
-       The authors need to reference and set the study within the context of the recently published WHO framework on scabies control - this can be found here: <https://www.who.int/publications/i/item/9789240008069>

**Response:** Thanks for your compliment. We have included the suggested reference in the introduction section of manuscript.

**Comment:** In addition to this there are some specific comments in relation to the introduction and typographical errors throughout the manuscript
The authors should go through the manuscript again as there are many typographical errors such as double full stops in lines 39, 55 and 164, standardize space between numbers and brackets for example in line 251 1430(91.3%) vs 1113 (94.0%), Table 1 “No” vs “No.” Line 474 whould say “Identifying” rather than “Indetifying”

**Response:** We appreciate the reviewer effort for reading the manuscript carefully and identifying all minor errors. We have accepted all suggestions and modified the manuscript. Thank you.

**Comment:** The ending of the sentence in lines 101-103 sounds incomplete, suggest replacing “with excellent safety” to “safely”

**Response:** The modification has been incorporated in the revised manuscript. Thank you.

**Comment:** The usage of reference 11 is incorrect, this is a letter in response to reference 10 and not a study about ivermectin MDA in Australia.

**Response:** We have made necessary corrections. Thanks for identifying the error.
