# Supplementary material for: Control of scabies in a tribal community using mass screening and treatment with oral ivermectin -A cluster randomized controlled trial in Gadchiroli, India
Source: PLoS Negl Trop Dis. 2021 Apr 16;15(4):e0009330. doi: 10.1371/journal.pntd.0009330 (PMC8081337; doi:10.1371/journal.pntd.0009330)
Supplement: Attachment — Submitted filename: Reviewer_Response_PNTD_D_20_01303.docx [file pntd.0009330.s009.docx]

**Response to Reviewer Comments: R2**

**Ref:** PNTD-D-20-01303

**Manuscript title:**  Control of scabies in a tribal community using mass screening and treatment with oral ivermectin -A cluster randomized controlled trial in Gadchiroli, India

We sincerely thank the reviewer for the comments. We have revised the manuscript accordingly. The reviewer has indicated the line numbers from the first version of the manuscript. A point to point response to each of the reviewer’s comment is given below. The revised manuscript (tracked changes file and clean file) along with the response to the reviewer’s comments are attached in the revised submission.

|  | **Reviewer’s comments** | **Authors Responses to reviewer’s comments** |
| --- | --- | --- |
| 1. 1 | Lines 126 to 134: The authors are requested to elaborate further on sample size calculation, with information about the rationale behind taking the prevalence of scabies to be 7%, and the size of the cluster considered while calculating DEFF from the given ICC of 0.2 | Thanks for the valuable comments.  The prevalence of scabies varies in tribal communities in India from 6.9-20.6% [Reference no 6&7] based on the published literature. Therefore, we considered the minimum estimated prevalence to get adequate sample size. This prevalence was also close to our expectation based on our experience of providing primary care in these villages over last several years. The size of the cluster considered while using the formula for calculating sample size was 250. The manuscript is now revised to reflect this. |
| 2. | Lines 132 to 134: The authors are requested to rephrase the sentence. | The sentence has been rephrased as per your suggestions. Thank you |
| 3. | Lines 344 and 345: Kindly rephrase the statement – it seems that the risk of persistence of scabies is more in the intervention arm from the mentioned sentence. | Thank you for pointing this error. The ARR listed for the disappearance was the ARR for the presence of scabies at two and 12 months. We have now corrected the ARR to reflect the rate of disappearance in the revised version |
| 4. | Lines 387 and 388: The claim of improved compliance with ivermectin needs to be bolstered with a reference. | We have now added a reference as per your suggestion. Thank you |
| 5. | Lines 392 to 395: The sentence needs re-framing to a passive voice | The sentence has been reframed in passive voice. Thanks for your suggestion. |
| 6. | Line 417: “Sixty nine (59.4%) referred scabies patients…” – It should be sixty or fifty-nine. | Typing error has been corrected. |
| 7. | Lines 450 and 451: An elaboration on the hypothesis would be appreciated. | We have now elaborated more on this point. We have also included two more references to support our argument.  Reference 27. Basti BD, Radhakrishnan S. Prevalence of dermatological manifestations among the tribal school children of South India. International Journal of Community Medicine and Public Health. 2016 Jul;3(7):1957-1962.  Reference 28. Bowen AC, Mahe A, Hay RJ, Andrews RM, Steer AC, Tong SY, et al. The Global Epidemiology of Impetigo: A Systematic Review of the Population Prevalence of Impetigo and Pyoderma. PLoS One. 2015 Aug 28;10(8):e0136789.  Thanks for your valuable suggestions. |
